# The Effect of Erythritol Injection in Decreasing of Abortion Rate in Local Breed Ewes

**DOI:** 10.1155/2023/8197703

**Published:** 2023-04-25

**Authors:** Maher Saber Owain, Mustafa Salah Hasan, Ali Ghazi Atiyah

**Affiliations:** ^1^Department of Surgery and Obstetrics, College of Veterinary Medicine, University of Tikrit, Tikrit, Iraq; ^2^College of Veterinary Medicine, University of Fallujah, Fallujah, Iraq; ^3^Taqaddam Foundation for Research and Strategic Studies, Fallujah, Iraq

## Abstract

This study designed to evaluate the effect of Erythritol injection in decreasing of abortion rate in local breed ewes. Fifty pregnant ewes from local breed aged 2–4 years with a history of abortion except G1, were fed ad libitum hay and grains with water. The study was carried out in Salah Aldein province at special farm at a period of July–November 2022. These animals were tests for brucella by using rose Bengal and ELISA at zero day for confirmation; these animals were divided into 5 groups: G1 was brucella −ve and pregnant at 60 days, G2 was brucella +ve and pregnant at 60 days, G3 brucella+ve pregnant animals and treated by antibiotics gentamicin 10%, 3 ml/animal for 3 days, G4 brucella +ve and pregnant and giving erythritol, 10 ml S/C of 10% solution (solve in water and glycerol), and G5 was brucella +ve, and all pregnant and giving Erythritol+ gentamycin 10%, 3 ml/animal for 3 days. The experiment takes 12 weeks. Blood was withdrawn at different times of experiment (0, 2 weeks and end of experiment). The seroprevalence of brucellosis was shown that all animals at G4 and G5 where seropositive after 14 days of experiment, at end of pregnancy the seropositivity were highly significantly in G4 and G5 as compared with another groups. The current results showed that percentages of abortion were higher in G2, followed by G3, while it has been reduced significantly in G4 and G1. In conclusion, Erythritol alone can decrease the rate of abortion by making the bacteria extracellular far from placenta and evading of infection by immunity and/or gentamicin injection. Also, erythritol can be used as elicit diagnosis of brucellosis in latent infected animals.

## 1. Introduction

Iraq has an estimated 6.6 million sheep and 1.3 million goats, which serve as a valuable source of milk and meat, as well as fiber production as well as a source of income and job security for those employed in the agricultural sector [1]. Unfortunately, disease is a major problem for the small ruminant sector in Iraq. Similar to many other endemic animal diseases, poor veterinary infrastructure makes it difficult to treat and control brucellosis and toxoplasmosis in Iraq [2, 3].

Three percent of the 3,255 cattle, one percent of the 1,060 sheep, four percent of the 845 goats, and two percent of the 404 mixed sheep and goats utilized in first-stage research from several regions in northern Iraq, including the Nineveh governorate, tested positive for the disease. Another research utilizing Brewer's card test found that 2.55 percent of studied goats and 0.78 percent of analyzed sheep were seropositive in the Mosul area [4]. However, there has been a surge in the number of seropositive animals in the city since then. Alhamada et al. [5] tested 91 flocks from Nineveh and found that 5.5% of the sheep and 5.3% of the goats tested positive for Brucella spp. antibodies using the Rose Bengal Test (RBT). In contrast, Kh Al-Hankawe et al. [6] found that c-ELISA seropositivity was present in 16.7% of cattle. In addition, while utilizing c-ELISA, KhAl-Hankawe et al. [7] found that 50% of buffaloes were seropositive. Although the causes of reproductive failure in small ruminants in Iraq are poorly understood, brucellosis and toxoplasmosis are two endemic illnesses that have been linked to decreased reproductive output and productivity [8, 9].

The placenta, which is contaminated with Brucella, is a crucial contact between the bacteria and the host. Brucella “endotoxin” generated in the placenta at levels as high as 10^13^ bacteria/gram of tissue [10] was formerly assumed to be responsible for the spontaneous miscarriages that characterize animal brucellosis. The presence of a growth-stimulatory substance, eventually identified as the 4-carbon sugar erythritol [11], in the fetal tissues was determined to be the cause of this abnormally high bacterial count. The inability of the *B. abortus* vaccine strain S19 to use erythritol as a carbon source was formerly thought to be directly linked to the virulence of the organism [12]. Recent research [13–15] suggests there may be a relationship between erythritol and virulence, albeit this correlation was not as strong as first thought.

Although the *Brucella melitensis *Rev-1 vaccine is thought to be the most effective one against brucellosis, its side effects make it less than ideal [16]. It is well known that the Rev.1 vaccine, a stable live attenuated strain of *B. melitensis* [17], given by the conjunctival route at recommended dosages, induces effective protection in sheep and goats against *B. melitensis*-related miscarriage [18]. The primary side effects of immunization include probable miscarriage in pregnant female animals receiving vaccinations, as well as potential Rev.1 genital or milk excretion [19, 20], shedding in feces has also been documented which can cause infection to another animals and humans.

This study designed to evaluate the protective effects of erythritol from abortion by brucella in sheep.

## 2. Materials and Methods

Fifty pregnant ewes from local breed aged 2–4 years with a history of abortion except G1, were fed ad libitum hay and grains with water. The study was done in Salah Aldein province at special farm at a period of July–November 2022.

Rose Bengal test: antibrucella antibodies can be detected in human and animal blood with the help of a slide agglutination test called the Rose Bengal. A micropipette was used to collect 75 L of blood on a rose Bengal plate. In order to guarantee a uniform solution, 25 L of the rose bengal-colored antigen was added to the serum after the container was vigorously agitated. After 4 minutes of mixing, the antigen and serum were ready to use. In this case, the outcome was seen after only 4 minutes. Clear clustering or agglutination was seen as a favorable response, while the absence of such behavior was seen as unfavorable. Prior to conducting the studies on the samples, controls were performed.

Commercially accessible tools were used to conduct the *i*-ELISA assay per the manufacturer's instructions (Mybiosource, USA).

These animals were tests for brucella by using rose Bengal and ELISA at zero day for confirmation, these animals were divided into the following 5 groups:

  G1 was brucella −ve and pregnant at 60 days
  G2 was brucella +ve and pregnant at 60 days
  G3 brucella +ve pregnant animals and treated by antibiotics gentamicin 10% (Vapco-jordon) 3 ml/animal for 3 days
  G4 brucella +ve and pregnant and giving erythritol (Bulk supplements, USA) 10 ml S/C of 10% solution (solve in water and glycerol).
  G5 was brucella +ve, and all pregnant, and giving Erythritol+ gentamycin 10% (Vapco-jordon) 3 ml/animal for 3 days

The experiment takes 12 weeks. Blood was withdrawn at different times of experiment (0, 2 weeks and end of experiment).

By pushing the slide surface tightly against the tissue, impression smear can be obtained from surfaces of newly sliced and wiped tissues, such as cotyledons. After they have dried in the air, they are fixed with heat. In addition, embryonic gastric juice, cotyledons, or lochia can be used to make smears that can be stained with the Giemsa stain or the stamp stain. Brucella organisms stain crimson in MZN-stained streaks, while most other bacteria stain blue.

### 2.1. Parameters

- Sonography done along the days of the experiment
- Rose Bengal which done at 0 and 2 weeks and at the end of pregnancy
- ELISA for detection IgG (Mybiosource, USA) which was carried out only at zero day for detection of infected animals

### 2.2. Statistical Analysis

Statistical analysis was carried out by using SPSS version 23.

## 3. Results

The sonar was carried out for all animals for detection of pregnancy.

After injection of erythritol, we noticed depression, decrease appetite and slight increase body temperature on animals for 12−24 hrs.

The seroprevalence of brucellosis was shown that all animals at G4 and G5 where seropositive after 14 days of experiment, at end of pregnancy the seropositivity were highly significantly in G4 and G5 as compared with another groups (Table 1).

**Figure 1:**
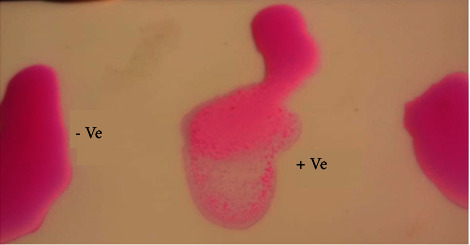
Rose Bengal positive and negative results.

**Figure 2:**
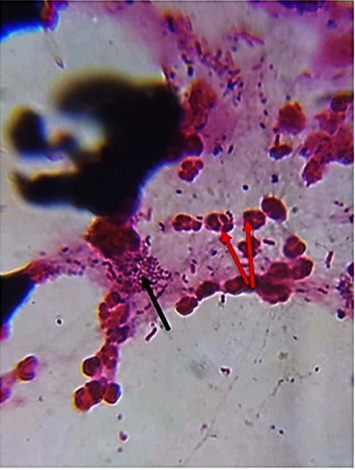
Impression smear for placenta shows Brucella (pointer black) with polymorphonuclear cells (red pointer).

The current results showed that percentages of abortion were higher significantly in G2, followed by G3, while it has been reduced significantly in G4 and G5 (Table 2).  Figure 1 shows positive and negative rose Bengal, while Figure 2 shows impression smear for placenta shows Brucella with polymorphonuclear cells.

## 4. Discussion

The Rose Bengal test (RBT) has been recommended as a suitable screening test for active infection at the national or local level for diagnosis of brucellosis in animals [21], It was confirmed as a useful screening test in livestock and humans [22]. The zoonotic illness brucellosis is caused by facultative intracellular-proteobacteria called *Brucella spp*., the presence of intracellularly make evading by immunity or antibiotics very hard [23]. Before anything else, it is important to note that although *B. ovis* and *B. canis* are unable to catabolize erythritol, they nonetheless manage to cause genital infections and abortions in sheep and dogs, respectively [24, 25]. Second, the erythritol-inhibiting effect of vaccination *B. abortus* S19, which may lead to genital infections and abortion in cattle [26], is an important consideration in animal health and animal husbandry. Third, Brucella may locate and even proliferate in cognate trophoblastic cell lines in the reproductive tracts of human and rodent hosts, but these hosts do not have significant erythritol concentrations [27, 28], these ideas may support our results.

The significant growth of brucella suppression due to lack of erythritol shown *in vitro* linked to the attenuation seen for certain *ery* gene mutants [29]. In addition to the tendency for external over intracellular growth, erythritol may have a role in this phenomenon since a significant colony of extracellular bacteria has been identified in the placentas of Brucella-infected cows [30]. We hypothesized that because erythritol stimulates cellular autophagy pathways, Brucella may be enticed to migrate out of its intracellular habitat in placental trophoblasts and proliferate in the extracellular milieu. When the bacteria escape the host cell, the host immunity may evade the infection. Also, we thought that animals in G5 have no abortion because of extracellular bacteria were killed with gentamycin immediately after infection, these also hypothesized by [31].

It was intriguing that, if erythritol could promote *B. melitensis* growth in broth culture, it did not also promote bacterial development inside macrophages [12]. Erythritol was found to be a suppressor of intracellular growth in earlier studies looking at how it affected intracellular *B. abortus* in cow trophoblasts, though these studies never compared growth with and without erythritol in the culture medium [32]. Because iron is crucial for the usage of erythritol and may be scarce within the Brucella intracellular compartment, it was postulated that in B. *abortus*, this intracellular growth deficiency in the presence of erythritol was caused [33, 34].

## 5. Conclusion

Erythritol alone can decrease the rate of abortion by making the bacteria extracellular far from placenta and evading of infection by immunity and/or gentamicin injection. Also, erythritol can be used as elicit diagnosis of brucellosis in latent infected animals.

## Figures and Tables

**Table 1:** Number of seropositive sheep among experimental animals.

Groups	Number of seropositive at day 0	Number of seropositive at day 14	Number of seropositive at end of pregnancy
G1	0	0	0
G2	0	0	7
G3	0	0	5
G4	0	10	10^*∗∗*^
G5	0	10	10^*∗∗*^

*P* values ^*∗∗∗∗*^*P* < 0.0001 Chi-square 55.17.

**Table 2:** Percentages of abortion among experimental groups.

Groups	Number of abortions	Percentages
G1	0	0
G2	4	40^*∗∗*^
G3	3	30^*∗*^
G4	1	10
G5	0	0

*P* values ^*∗*^*P* < 0.04 Chi-square 9.821.

## Data Availability

The data used to support the findings of this study are included within the article.
